# OTUB1-catalyzed deubiquitination of FOXM1 facilitates tumor progression and predicts a poor prognosis in ovarian cancer

**DOI:** 10.18632/oncotarget.9160

**Published:** 2016-05-04

**Authors:** Yiqin Wang, Xianrong Zhou, Midie Xu, Weiwei Weng, Qiongyan Zhang, Yusi Yang, Ping Wei, Xiang Du

**Affiliations:** ^1^ Department of Pathology, Obstetrics and Gynecology Hospital of Fudan University, Shanghai 200044, China; ^2^ Department of Pathology, Fudan University Shanghai Cancer Center, Shanghai 200032, China; ^3^ Department of Pathology, Shanghai Medical College, Fudan University, Shanghai 200032, China; ^4^ Institute of Pathology, Fudan University, Shanghai 200032, China; ^5^ Institute of Biomedical Sciences, Fudan University, Shanghai 200032, China; ^6^ Cancer Institute, Fudan University Shanghai Cancer Center, Shanghai 200032, China

**Keywords:** OTUB1, FOXM1, ovarian carcinoma, ubiquitination, deubiquitination

## Abstract

Ubiquitination is essential for regulation of cell physiology, protein stability, and signal transduction [1]. Its dysregulation is an important factor in many diseases, including cancer. We explored the potential OTUB1-catalyzed deubiquitination of FOXM1, a transcription factor linked to carcinogenesis, and the biological consequence of that interaction in ovarian cancer. We found that FOXM1 is ubiquitinated by multiple polyUb chains and targeted for proteosomal degradation in a reaction dependent on its ubiquitination-required KEN box. Additionally, the OTUB1 N-terminus and catalytic triad bind to FOXM1, specifically catalyzing cleavage of the K48-specific ubiquitin linkage from FOXM1. Moreover, OTUB1-FOXM1 interaction drives tumor progression and OTUB1 expression predicts a poor prognosis in ovarian cancer. Our study suggests that inhibiting OTUB1-FOXM1 interaction is a potential new avenue for ovarian cancer therapy.

## INTRODUCTION

In the process of ubiquitination, ubiquitin (Ub), a 76 amino acid protein, is conjugated to the lysine residues of specific substrate proteins via the transfer from E1 activating enzyme to E2 conjugating enzyme and subsequent binding catalyzed by E3 ligase [2]. Ub contains seven lysine residues for conjugation, each of which can form a specific polyubiquitin (polyUb) chain with distinct functions. The classic linkages such as K48- and K11-lead to proteosomal degradation of the substrate protein while K63-functions in signal transduction [3]. Ubiquitination is reversed by the deubiquitinating enzymes (DUBs), which hydrolyze the isopeptides between the substrates and Ub or directly cleave the polyUb chains [4]. DUBs enable reciprocal biological functions by catalyzing deubiquitination of crucial proteins [2].

FOXM1 is a Forkhead family protein reported to promote tumorigenesis [5, 6]. FOXM1 dysregulation leads to uncontrolled cell growth and epithelial-mesenchymal transition [7, 8] in somatic malignancies including breast [9], liver [10], lung [11], and ovarian carcinomas [12]. Although FOXM1 is a predicted target of the ubiquitin-proteosome system [13–15], little is known about either its degradation-related domains or its affinity for different polyUb chains.

OTUB1 is one of the most abundant DUBs in somatic cells [16] and a member of the ovarian tumor (OTU) protein superfamily [17]. OTUB1 is implicated in the NF-κβ and TGF-β signaling pathways [18] by increasing c-IAP2 [19], phosphorylated SMAD2/3 [18], and TRAF3/6 [20]. Many proteins downstream of OTUB1, such as p53 [21, 22], ERα [23, 24] and SMAD2/3 [18, 25], are also downstream targets of FOXM1, suggesting a possible link between the two genes. Our study aimed to explore their potential connection and underlying molecular mechanism that might contribute to the tumor progression and prognosis in ovarian cancer.

## RESULTS

### OTUB1 interacts with FOXM1 *in vitro* and *in vivo*

**Figure 1:**
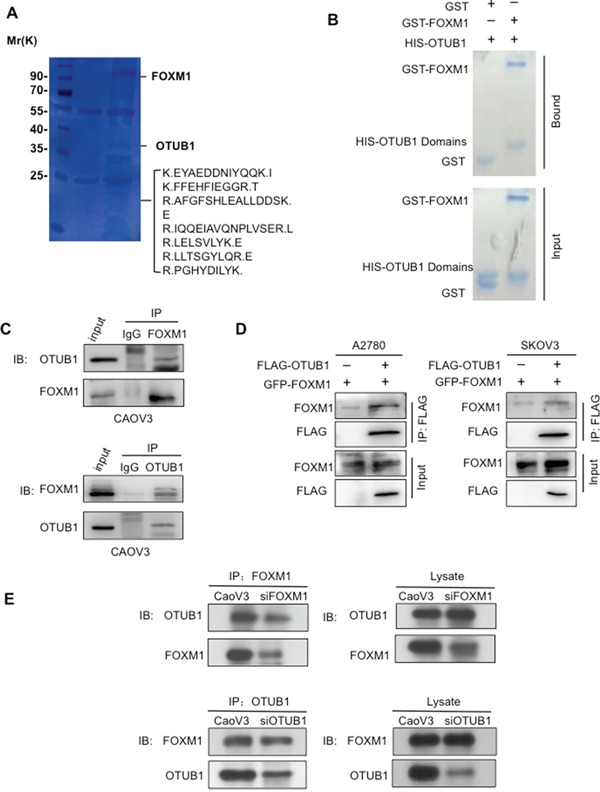
OTUB1 interacts with FOXM1 *in vitro* and *in vivo* **A.** The LCMS/MS detected sequences of OTUB1 in the FLAG-FOXM1 IP complex. **B.** GST-pulldown showed purified GST-FoxM1 but not GST binding to the purified HIS-OTUB1 protein. **C.** The IP results suggest reciprocal interactions between endogenous OTUB1 and FOXM1 in CAOV3 cells. 15% of cell lysates were used as input. **D.** The co-IP results indicate that ectopic FLAG-OTUB1 interacted with GFP-FOXM1 in A2780 and SKOV3 cells. 15% of cell lysates were used as input. **E.** The IP results of indicate that either knockdown of OTUB1 or FOXM1 by siRNA reduced the binding FOXM1 or OTUB1 in CAOV3 cells. 15% of cell lysates were used as input.

First, the LC-MS/MS analysis of purified FLAG-FOXM1 containing complex revealed peptide sequences of OTUB1 (Figure 1A), suggesting that OTUB1 binds to FOXM1 (Supplementary Figure 1A, Supplementary Table 3). The interaction between FOXM1 and OTUB1 was confirmed by co-IP in 293T cells (Supplementary Figure 1B). Moreover, glutathione S-transferase (GST)-pulldown showed that purified HIS–OTUB1 was specifically bound by purified GST–FOXM1 protein, but not GST alone (Figure 1B). Taken together, these data suggest that OTUB1 and FOXM1 directly bind to each other *in vitro*.

We next detected OTUB1 mRNA and protein levels in a panel of cell lines and chose the candidate A2780 and SKOV3 cells for OTUB1 overexpression and CAOV3 cells for knockdown (Supplementary Figure 1C). We noticed that the protein levels but not the mRNA levels of OTUB1 and FOXM1 were correlated in cell lines (Supplementary Figure 1C-1D). Then, we found the interactions between endogenous OTUB1 and FOXM1 by IP assay in CAOV3 cells (Figure 1C), as well as between ectopically expressed OTUB1 and FoxM1 by co-IP in A2780 and SKOV3 cells (Figure 1D). Moreover, siRNA knockdown of OTUB1 or FOXM1 reduced the endogenous binding of FOXM1 or OTUB1, respectively, in CAOV3 cells (Figure 1E). Taken together, these results strongly suggest that OTUB1 interacts with FOXM1.

### OTUB1 and FOXM1 co-expression are associated with poor ovarian cancer prognosis

**Figure 2:**
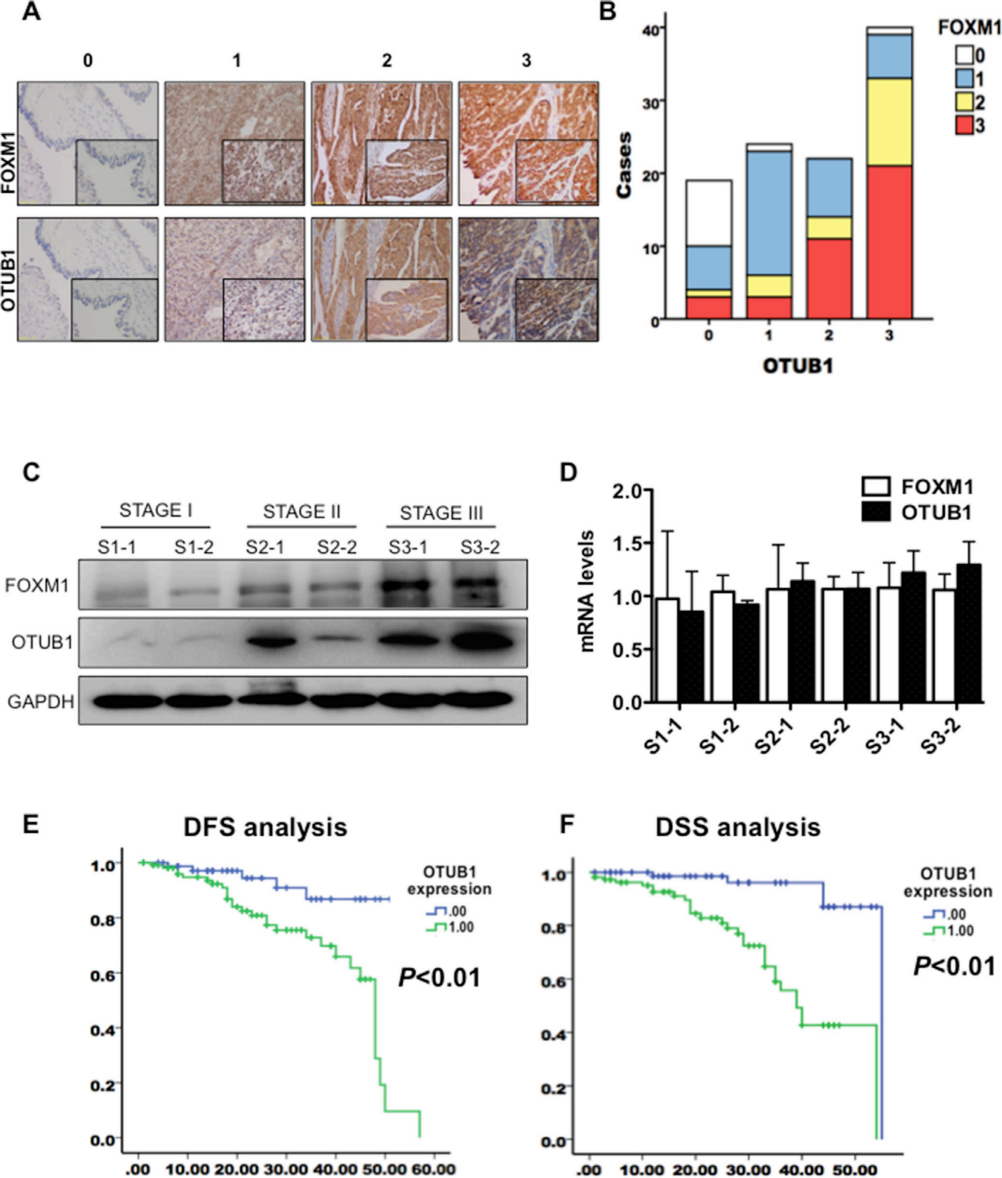
OTUB1 co-localizes with FOXM1 and elevation of OTUB1 predicts poor ovarian cancer prognosis **A.** Representative images of score 0-3 of OTUB1 and FOXM1 expression detected by immunochemistry. **B.** Pearson correlation analysis of immunostaining scores of OTUB1 and FOXM1 (*r*=0.610, *p*<0.01). **C.** IB results of OTUB1 and FOXM1 in 25 tissues of ovarian carcinoma at Stage I, II and III. **D.** RT-qPCR results of OTUB1 and FOXM1 in 25 tissues of ovarian carcinoma at Stage I, II and III. **E.** Kaplan-Meier disease-free survival (DFS) curves of patients with different expressions of OTUB1 in ovarian cancer (Low vs. High). **F.** Kaplan-Meier disease-specific survival (DSS) curves of patients with different expressions of OTUB1 in ovarian cancer (Low vs. High).

To explore the correlation between OTUB1 and FOXM1 in ovarian cancer, we analyzed the immunostaining of OTUB1 and FOXM1 protein in samples from 200 ovarian cancer patients. Both OTUB1 and FOXM1 were highly expressed in tumor lesions relative to paratumorous tissues (Figure 2A) with statistically significant correlation (Figure 2B, *r*=0.610, *p*<0.01). OTUB1 and FOXM1 expression were consistently correlated in immunoblotting results of total protein extracted from 25 ovarian carcinoma tissue samples (Supplementary Figure 2B, *r*=0.448, *p*=0.025). Furthermore, both proteins increased with the progression of malignant staging (Figure 2C), while the mRNA levels remained irrelevant (Figure 2D).

Next, we elucidated the clinical significance of OTUB1 and FOXM1 protein in 200 tissues of ovarian cancer. OTUB1 expression gradually increased with the advance of stage (Supplementary Figure 2C), tightly correlated with FIGO staging, lymph node metastasis, tumor size, and recurrence (*p*<0.05, Table 1). However, ages and histological subtypes were not correlated with OTUB1 expression (Table 1). Kaplan-Meier analysis using the log-rank test suggested that patients with high expression of both OTUB1 (n=112, score 2-3, see Materials and Methods) and FOXM1 (n=93, score 2-3, see Materials and Methods) had significantly shorter disease free survival (DFS) (OTUB1: *p*<0.01, Figure 2E and Supplementary Figure 2D) and disease specific survival (DSS) (P<0.05, Figure 2F and Supplementary Figure 2D) than low expressing patients (OTUB1: n=88, score 0-1; FOXM1: n=107, score 0-1). A univariate Cox analysis implied that FIGO staging, lymph node metastasis, FOXM1, and OTUB1 correlated with survival (Supplementary Tables 4-5). Multivariate analysis using the Cox proportional hazard model demonstrated that both OTUB1 and FOXM1 were independent risk factors for DSS (*p*<0.05), but only OTUB1 was an independent risk factor for DFS (*p*<0.05) (Supplementary Tables 4-5). Together, our results underscore the clinical relevance of OTUB1 in ovarian cancer progression and suggest OTUB1 as a novel therapeutic target and prognostic biomarker for the disease.

**Table 1:** Correlation between clinicopathological features and expression of OTUB1

Clinicopathological Features		N	%	OTUB1 Expression				Rho value	*P* value
				0	1	2	3		
All cases		200	100	31	57	43	69		
Age (Average 37.3±6.57years)	<40	88	44.00	18	25	17	28	0.098	0.169
	≥40	112	56.00	13	32	26	41		
Tumor size	<1cm	84	42.00	17	28	17	22	0.177	0.012*
	≥1cm	116	58.00	14	29	26	47		
FIGO Stage	I	96	48.00	26	44	13	13	0.548	<0.001*
	II	58	27.00	4	10	16	28		
	III, IV	46	23.00	1	3	14	28		
	Serous	55	27.50	6	11	9	29		
Histological Types	Endometrioid	60	30.00	9	16	16	19	−0.126	0.075
	Clear cell	67	38.50	13	24	14	16		
	Mucinous	18	9.00	3	6	4	5		
Opposite ovary involvement	-	113	56.50	23	36	22	32	0.199	0.005*
	+	87	43.50	8	21	21	37		
Tumor cells in peritoneal fluid	-	132	61.00	26	47	45	25	0.407	<0.001*
	+	68	39.00	44	10	9	44		
Fallopian tube involvement	-	114	57.00	26	37	20	31	0.269	<0.001*
	+	86	43.00	5	20	23	38		
Peritoneal implantation	-	152	76.00	29	54	29	40	0.368	<0.001*
	+	48	24.00	2	3	14	29		
Lymph node metastasis	-	174	87.00	31	55	35	53	0.280	<0.001*
	+	26	13.00	0	2	8	16		
Remote metastasis	-	198	99.00	31	57	43	67	0.119	0.094
	+	2	1.00	0	0	0	2		
Recurrence	-	149	83.00	30	53	29	37	0.410	<0.001*
	+	51	17.00	1	4	14	32		

Abbreviation: N, number of patients

* P<0.05

### OTUB1 elevates FOXM1 protein levels via the proteosomal degradation pathway

**Figure 3:**
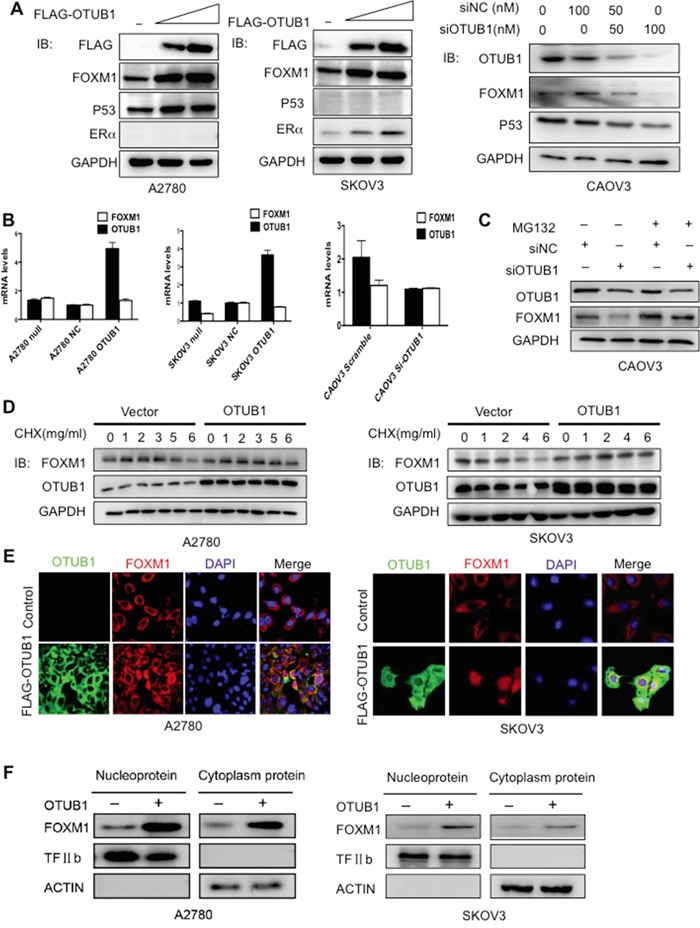
OTUB1 positively regulates FOXM1 post-transcriptionally in cytoplasm **A.** Immunoblotting results of FOXM1 and OTUB1 in A2780 and SKOV3 cells transiently transfected with indicated plasmids and in CAOV3 cells with siOTUB1. **B.** RT-qPCR results of FOXM1 and OTUB1 in A2780 and SKOV3 cells transiently transfected with indicated plasmids and in CAOV3 cells with siOTUB1. **C.** CAOV3 cells were transfected with scramble or siOTUB1 for 48 h. MG132 (10 μM) was added for 3 hours before harvesting. Lysates were immunoblotted with indicated antibodies. **D.** A2780 and SKOV3 cells were transfected with indicated plasmids for 48 h. CHX (50 μg/mL) then was added at 0 h, 1 h, 2 h, 4 h, and 6 h. Lysates were collected at indicated time points and immunoblotted with indicated antibodies. **E.** The immunofluorescence results of cellular localization of FOXM1 and FLAG-OTUB1 in A2780 and SKOV3 cells. **F.** The western blot results of nuclear/cytoplasm fractioned FOXM1 protein levels in OTUB1-overexpressing A2780 and SKOV3 cells.

Next, we observed that overexpression of OTUB1 elevated the protein levels of FOXM1 in the wild-type p53 (+)/ERα (−) A2780 and the p53 (−)/ERα (+) SKOV3 cells, suggesting that OTUB1 increases FOXM1 in a p53/ERα independent manner (Figure 3A). siRNA knockdown of OTUB1 decreased FOXM1 protein level in CAOV3 cells in a dose-dependent manner (Figure 3A). In contrast, FOXM1 mRNA level was unchanged (Figure 3B). Furthermore, the proteasome inhibitor MG132 rescued the reduction of FOXM1 caused by knockdown of OTUB1 in CAOV3 cells (Figure 3C), whereas overexpression of OTUB1 in A2780 and SKOV3 moderately prolonged the half-life of FOXM1 in the presence of the protein biosynthesis inhibitor CHX (Figure 3D and Supplementary Figure 3B). Finally, the immunofluorescence and the nuclear/cytoplasm fractioned immunoblotting results show that overexpression of OTUB1 elevated FOXM1 protein levels both in cytoplasm and nucleus (Figure 3E-3F and Supplementary Figure 3C). These results show that OTUB1 elevates FOXM1 by inhibiting its proteasome-mediated degradation.

### OTUB1 stabilizes FOXM1 by disassembling the K48-specific linkage

**Figure 4:**
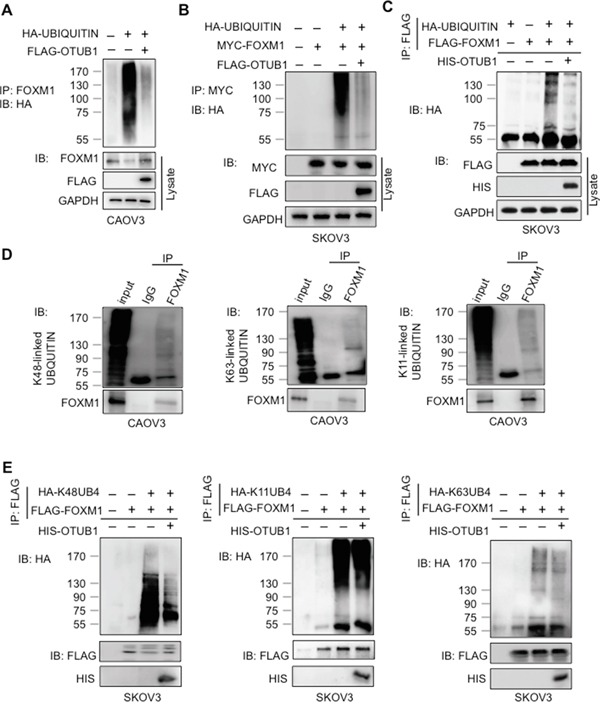
OTUB1 suppresses FOXM1 ubiquitination by cleaving the K48-specific polyUb chain **A-B.** The *in vivo* ubiquitination results show that overexpression of FLAG-OTUB1 suppressed the ubiquitination of endogenous FOXM1 in CAOV3 (A) and exogenous MYC-FOXM1 in SKOV3 cells (B). **C.** The *in vitro* deubiquitination results that the ubiquitinated FLAG-FOXM1 was deubiquitinated *in vitro* by purified HIS-OTUB1. **D.** Lysates of CAOV3 cells were subjected to IP with anti-K48, anti-K63 or anti-K11 antibodies followed by IB with anti-FOXM1 antibody. 15% of cell lysates were used as input. **E.** The *in vitro* deubiquitination assays showed that the K48-specific other than K-11 or K-63 ubiquitinated FOXM1 was deubiquitinated *in vitro* by purified HIS-OTUB1.

The observed modulation and direct contact between OTUB1 and FOXM1 prompted us to ask whether OTUB1 affects FOXM1 ubiquitination. The *in vivo* ubiquitination assay showed that overexpression of OTUB1 drastically reduced the ubiquitination of both endogenous and exogenous FOXM1 (Figure 4A-4B and Supplementary Figure 4A), and knockdown of OTUB1 increased ubiquitinated FOXM1 (Supplementary Figure 4B). Furthermore, purified recombinant HIS-OTUB1 markedly reduced ubiquitinated FOXM1 *in vitro*, suggesting direct deubiquitination of FOXM1 (Figure 4C and Supplementary Figure 4C).

We conducted IPs and found that FOXM1 was ubiquitinated by classic polyUb chains including K48-, K63-, and K11-specific linkages in CAOV3 cells (Figure 4D). OTUB1 specifically disassembled the K48-linked polyUb chains, rather than the K63 or K11-specific linkages, from FOXM1 (Figure 4E). These results suggest that OTUB1 directly deubiquitinates FOXM1 by canonically cleaving the K48-specific linkage.

### The OTUB1 and FOXM1 domains essential for DUB function

**Figure 5:**
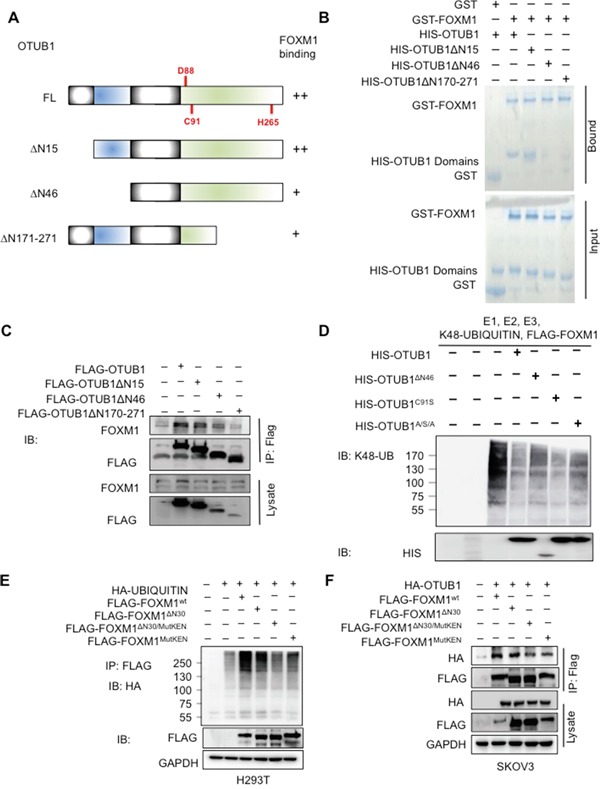
The domains of OTUB1 and FOXM1 essential for DUB function **A.** The structure of full-length OTUB1 and its mutants. The key residues and domains of OTUB1 were shown. **B.** The GST-pulldown shows that the N-terminal 16-46aa and C-terminus of OTUB1 bound to GST-FOXM1 *in vitro.* **C.** The co-IP shows that the N-terminal 16-46aa and C-terminus of OTUB1 interact with FOXM1 *in vivo.* 15% of cell lysates were used as input. **D.** The *in vitro* ubiquitination assay shows that the DUB activity on FOXM1 was attenuated in both OTUB1^ΔN46^ and OTUB1^A/S/A^. **E.** The *in vivo* ubiquitination results show that the polyubiquitination of FOXM1^MutKEN^ and FOXM1^ΔN30/MutKEN^ was reduced compared with full-length FOXM1 and FOXM1^ΔN30^ in H293T cells. **F.** The co-IP results indicate that the affinities of FOXM1^MutKEN^ and FOXM1^ΔN30/MutKEN^ for OTUB1 were attenuated compared with full-length FOXM1 and FOXM1^ΔN30^ in SKOV3 cells. 15% of cell lysates were used as input. OTUB1^A/S/A^: OTUB1^D88A/C91S/H265A^.

Since OTUB1 contains a proximal Ub binding site at the N terminus and a catalytic center containing Asp88, Cys91, and His265 [26, 27], we generated truncation or mutants of OTUB1 to map the crucial domains for deubiquitinating FOXM1 (Figure 5A). GST-pulldown and *in vivo* co-IP showed that depletion of N-terminal 1-46aa (OTUB1^ΔN46^) or 171-271aa (OTUB1^ΔN171-271^) but not N-terminal 1-15aa (OTUB1^ΔN15^) reduced the affinity for FOXM1 (Figure 5B-5C). OTUB1^ΔN15^ retained DUB activity for FOXM1 while OTUB1^ΔN46^ disrupts DUB activity *in vivo* and *in vitro* (Supplementary Figure 5A and 5B), suggesting the necessity of N-terminal 16-46aa to the DUB activity of OTUB1. OTUB1^C91S^ and OTUB1^D88A^ retained DUB function while the double substitutions (OTUB1^C91S/H265A^) and the triple substitutions (OTUB1^D88A/C91S/H265A^, OTUB1^A/S/A^) gradually lost DUB function (Supplementary Figure 5C). Moreover, OTUB1^A/S/A^ and OTUB1^ΔN46^ failed to suppress K48-specific ubiquitination of FOXM1 *in vitro* (Figure 5D), implying that the integrity of the catalytic triad of OTUB1 is essential to its DUB activity.

Since the N terminus of FOXM1 (N-terminal 2-30aa and the KEN box at N-terminal 207-209aa) is implicated in the process of degradation [13], we constructed truncated and mutant FOXM1 (FOXM1^ΔN30^, FOXM1^MutKEN^ and FOXM1^MutKEN/ΔN30^). The *in vivo* ubiquitination assay suggested that FOXM1^ΔN30^ was normally ubiquitinated whereas polyubiquitinated FOXM1^MutKEN^ and FOXM1^MutKEN/ΔN30^ were reduced (Figure 5E). Co-IP assay showed that the N-terminal 1-30aa deletion (FOXM1^ΔN30^) retained full-length affinity to OTUB1 while the binding of OTUB1 to both KEN box mutants (FOXM1^MutKEN^ and FOXM1^MutKEN/ΔN30^) was reduced (Figure 5F). These results suggest that the KEN box is more involved in OTUB1-FOXM1 deubiquitination activity than the N-terminal 30aa.

### OTUB1 plays a critical role in ovarian cancer pathogenesis by deubiquitinating FOXM1

To study the biological effects of OTUB1 on ovarian cancer, we first confirmed the oncogenic functions of OTUB1 on cell proliferation *in vitro* (Supplementary Figure 6A-6D), which was further identified in the *in vivo* xenograft models (Supplementary Figure 6G); OTUB1 also promoted cell migration and invasion *in vitro* (Supplementary Figure 6E-6F).

**Figure 6:**
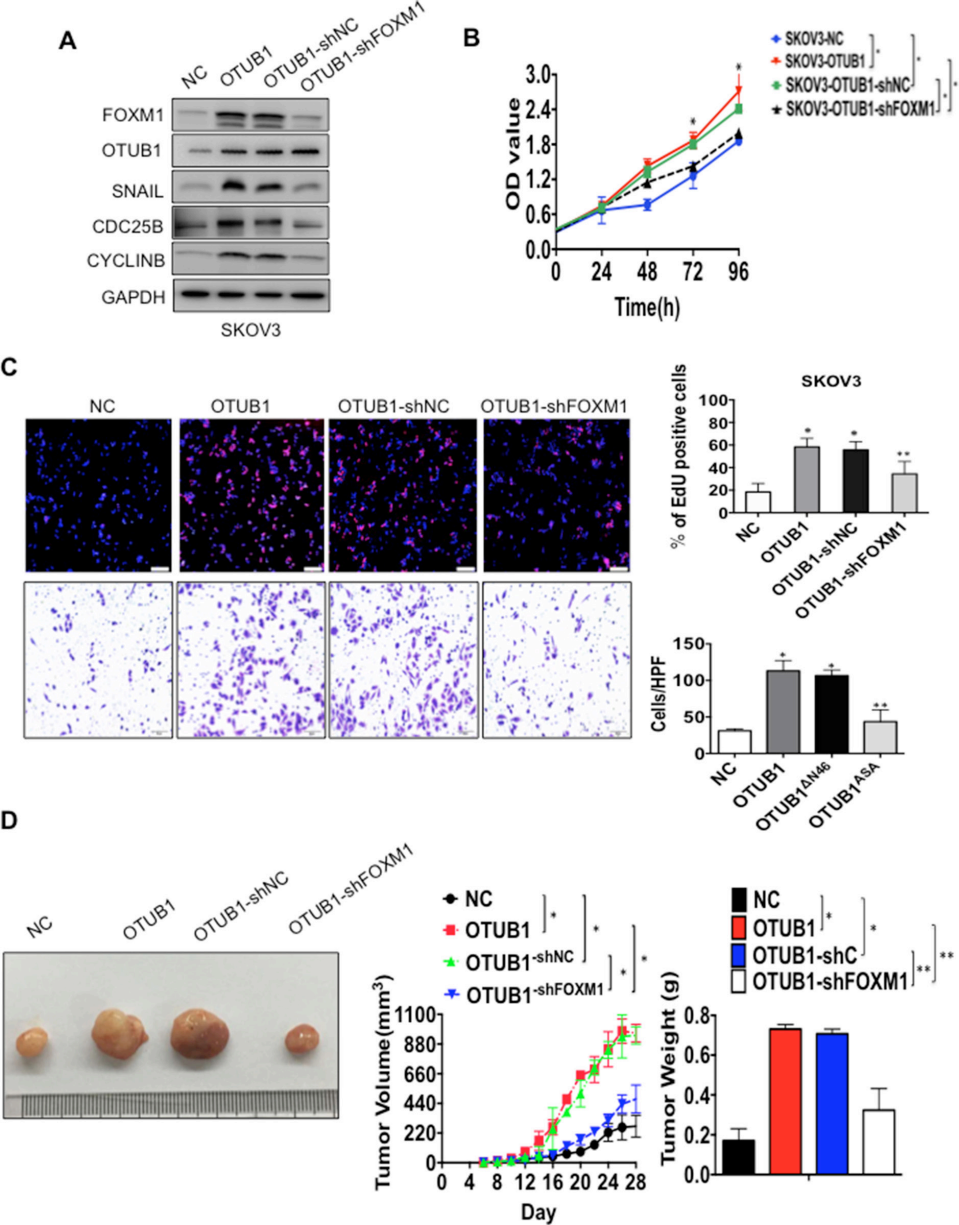
OTUB1 exerts oncogenic functions in a FOXM1-meidated manner **A.** The western blotting results showed the expression of downstream targets of FOXM1 SNAIL, CDC25B and CyclinB in NC, OTUB1, OTUB1-shC and OTUB1-shFOXM1 infected SKOV3 cells. **B.** The CCK8 results of NC, OTUB1, OTUB1-shC and OTUB1-shFOXM1 infected SKOV3 cells. *: *p*<0.01. **C.** Representative images of EdU immunofluorescence and transwell assay in NC, OTUB1, OTUB1-shC and OTUB1-shFOXM1 infected SKOV3 cells. The percentage of EdU positive cells were graphed under 100× and calculated under 200× magnification. The penetrated cells were graphed under 200× and counted under 400× magnification. *: *p*<0.01. **: *p*<0.05. **D.** The xenograft tumorigenesis results of NC, OTUB1, OTUB1-shC and OTUB1-shFOXM1 infected SKOV3 cells. Tumors were photographed and the speed of tumor growth was illustrated as curves, and the weights of tumors were calculated and analyzed. *: *p*<0.01. **: *p*<0.05.

Then we turned to investigate whether OTUB1 exerted its oncogenic functions in a FOXM1-mediated manner. The immunoblotting results suggested that overexpression of OTUB1 could elevate the expressions of the downstream targets of FOXM1 such as SNAIL, CDC25B and CyclinB, while knockdown of FOXM1 drastically reduce the expressions of these genes in spite of OTUB1 overexpression (Figure 6A). The CCK8, EdU and tranwell assays showed that knockdown of FOXM1 potently attenuated the enhanced SKOV3 cell proliferation (*p*<0.01, Figure 6B-6C) and invasion caused by overexpression of OTUB1 (*p*<0.01, Figure 6C). Similarly, the *in vivo* xenograft model showed that overexpression of OTUB1 could accelerate the tumor growth speed and increase the tumor weight, which were both suppressed by knockdown of FOXM1 in spite of OTUB1 overexpression (*p*<0.01, Figure 6D). All these data suggest that OTUB1 exerts its oncogenic functions in a FOXM1-mediated manner.

**Figure 7:**
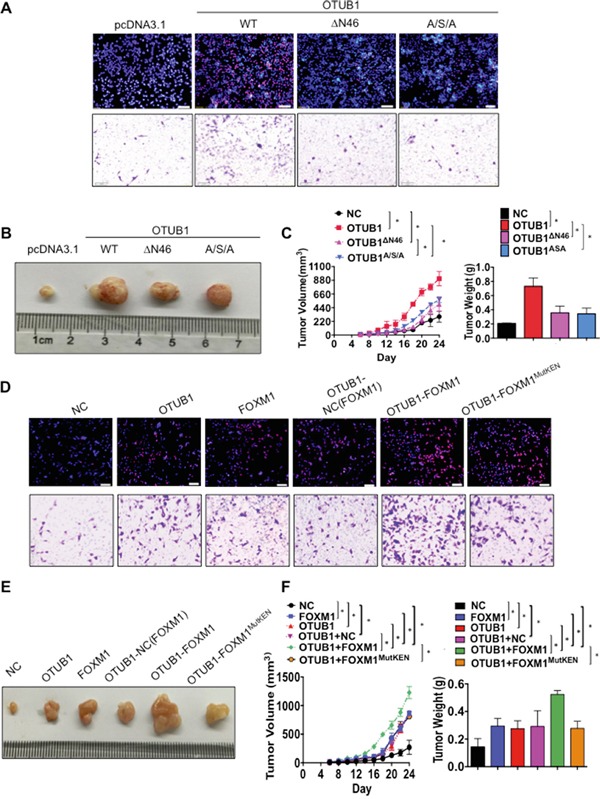
OTUB1 exerts oncogenic functions via suppression of FOXM1 ubiquitination **A.** Representative images of EdU immunofluorescence and transwell assay in NC, OTUB1, OTUB1^ΔN46^ and OTUB1^A/S/A^ infected SKOV3 cells. The percentage of EdU positive cells were graphed under 100× and calculated under 200× magnification. The penetrated cells were graphed under 200× and counted under 400× magnification. *: *p*<0.01. **: *p*<0.05. **B-C.** The xenograft tumorigenesis results of NC, OTUB1, OTUB1^ΔN46^ and OTUB1^A/S/A^ infected SKOV3 cells. Tumors were photographed (B) and the speed of tumor growth was illustrated as curves, and the weights of tumors were calculated and analyzed (C). *: *p*<0.01. **D.** Representative images of EdU immunofluorescence and transwell assay in NC, OTUB1, FOXM1, OTUB1-NC(FOXM1), OTUB1-FOXM1 and OTUB1-FOXM1^MutKEN^ infected SKOV3 cells. The percentage of EdU positive cells were graphed under 100× and calculated under 200× magnification. The penetrated cells were graphed under 200× and counted under 400× magnification. *: *p*<0.01. **E-F.** The xenograft tumorigenesis results of NC, OTUB1, FOXM1, OTUB1-NC(FOXM1), OTUB1-FOXM1, and OTUB1-FOXM1^MutKEN^ infected SKOV3 cells (E). Tumors were photographed and the speed of tumor growth was illustrated as curves, and the weights of tumors were calculated and analyzed (F). *: *p*<0.01. FOXM1^MutKEN^: FOXM1^K207A/E208A/N209A^.

Next we elucidated the potential influence of essential domains of OTUB1 on the functions of OTUB1. The CCK8, EdU and transwell assays showed that overexpression of OTUB1^ΔN46^ and OTUB1^A/S/A^ did not stimulate SKOV3 cell proliferation (*p*<0.01, Supplementary Figure 7A-7B and Figure 7A) or invasion (*p*<0.01, Figure 7A and Supplementary Figure 7B) as potently as full-length OTUB1. Besides, overexpression of OTUB1^ΔN46^ or OTUB1^A/S/A^ failed to elevate tumor weight or to accelerate tumorigenesis as much as OTUB1 wild type (*p*<0.01, Figure 7B-7C). These results suggest that the N terminus and the catalytic triad are vital to the biological functions of OTUB1.

Furthermore, we explored the essentiality of OTUB1-FOXM1 interaction to the biological function of OTUB1 in ovarian cancer. CCK8 and EdU assay showed that overexpression of either OTUB1 or FOXM1 stimulates SKOV3 cell proliferation and invasion, which could be enhanced by co-overexpression of OTUB1 with FOXM1 wild type but not FOXM1^MutKEN^ (*p*<0.01, Figure 7D and Supplementary Figure 7D-7E). Moreover, overexpression of either OTUB1 or FOXM1 accelerated tumorigenesis and elevated tumor weight, which could be further enhanced by co-overexpression of OTUB1-FOXM1 but not OTUB1-FOXM1^MutKEN^ (*p*<0.01, Figure 7E-7F). These data indicate that the OTUB1-FOXM1 interaction is essential to the proliferation and invasion of ovarian cancer.

**Figure 8:**
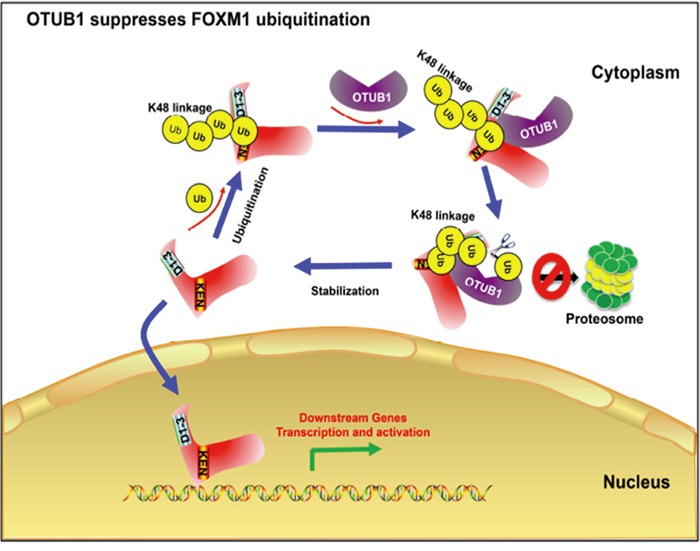
A Schematic of the mechanism how OTUB1 regulates FOXM1 FOXM1 is ubiquitinated by polyUb chains in cytoplasm leading to a proteasome-mediated degradation, which needs the conformation of its N-terminal 30aa and KEN box. OTUB1 is recruited and binds to FOXM1 and then recognizes the K48-specific linkage. Once confirming the linkage, OTUB1 binds to Ub via both the N-terminal proximal Ub binding site (N-terminal 16-46aa) and the OTU domain, cleaving the linkage and stabilize FOXM1. Consequently, more FOXM1 enters the nucleus to exert its function to activate the transcription of downstream genes.

Finally we analyzed the mechanism of OTUB1-FOXM1 interaction in ovarian cancer (Figure 8). FOXM1 is ubiquitinated by polyUb chains in the cytoplasm leading to its proteasome-mediated degradation, which requires the KEN box. OTUB1 binds to FOXM1 and then recognizes the K48-specific linkage. After confirming the linkage, OTUB1 binds to Ub via both the N-terminal proximal Ub binding site (N-terminal 16-46aa) and the catalytic triad, cleaving the linkage and stabilizing FOXM1. Consequent FOXM1 accumulation increases transcription of downstream genes.

## DISCUSSION

Our study shows that OTUB1, highly expressed in ovarian cancer, binds to FOXM1 and cleaves the K48-linked polyUb chain, stabilizing FOXM1 to promote tumorigenesis and tumor progression. Therefore, the OTUB1-FOXM1 interaction plays a critical role in ovarian tumorigenesis and tumor progression.

An elegant study has revealed that the K11-specific linkage is preferred by APC/C E3 ligase [28]. CDH1, a member of the APC/C family, has been suggested to be the E3 ligase of FOXM1 by Park et al. and Laoukili et al. [13, 14]. The combination of N-terminal 30aa covering domain D1-3 and N-terminal 207-209aa (KEN box) appear essential to Cdh1-mediated degradation, as N-terminal 30aa deletion or mutation of KEN box abolished stabilization of FOXM1 [13, 14]. Our data show that FOXM1 is ubiquitinated by several polyUb linkages, and OTUB1 specifically disassembles the K48-specific linkage. In contrast to previous observation in CDH1 [13, 14], the KEN box alone affected the ubiquitination of FOXM1 as well as the affinity for OTUB1 in our study. Furthermore, OTUB1 failed to enhance the function of FOXM1^MutKEN^. The reduced affinity of FOXM1^MutKEN^ for OTUB1 could be a defect in the direct interaction of FOXM1 and OTUB1, or more likely, it could be a direct consequence of reduced binding of OTUB1 due to defective K48Ub linked polyubiquitination of FOXM1^MutKEN^. Based on these findings, we deduce that different Ub linkages could induce variability of conformation. One conformation centered with the KEN box is recognized and favored by OTUB1-mediated K48-specific deubiquitination.

The N terminus of FOXM1 and its interaction with CDH1 and UBC9 [29] has been reported to be essential to its sumoylation and subsequent inhibition, suggesting that the N terminus of FOXM1 is crucial to the degradation of FOXM1. Although we found it was the KEN box that refer to the ubiquitination of FOXM1 in our study, there still lie the possibilities that other motifs at the N terminus of FOXM1 might contribute to the translational modification of FOXM1. Indeed, Myatt et al. found that the sumoylation sites of FOXM1 are scattered within the N-terminal 201-500 aa of FOXM1 around the KEN box [29], and OTUB1 suppresses sumoylation in cells [18, 27], it would be interesting to explore the interaction between OTUB1 and other domains at the N terminus of FOXM1 and the effect of OTUB1 on FOXM1 sumoylation.

Besides the canonical K48-specific catalytic pathway [30, 31], OTUB1 reportedly has a non-canonical mechanism of interference with the conjugation of Ub to E2 or the transfer of Ub from E2 to E3 [32]. Our results suggest that OTUB1 requires its catalytic domain and the N-terminal 16-46aa to suppress FOXM1 ubiquitination *in vivo* and *in vitro*. The integrity of the catalytic triad is crucial as the double or triple point mutants lost their DUB abilities incrementally. The N-terminal 16-46aa of OTUB1 contains the proximal Ub binding site [31], the depletion of which drastically attenuated the DUB activity as well as biological functions of OTUB1. Recent studies have found that the OTU deubiquitinases recognize the specific linkage depending on their proximal Ub binding sites [33]. The proximal binding site of OTUB1 forms an α-helix to interact with free Ub, the charged E2~Ub, and the discharged E2, regulating the classical DUB activity of OTUB1, suggesting cooperation of canonical and non-canonical pathways [16]. Although our study illustrates that OTUB1 suppresses FOXM1 in a canonical catalytic manner, OTUB1 may still bind E2~Ub via its proximal helix to regulate the stability of FOXM1.

A relatively new oncogene, conflicting roles of OTUB1 have been described in colon cancer and lung tumor cell lines [22, 34]. Our results indicate that OTUB1 exerts oncogenic activities in ovarian cancer and its expression is tightly correlated with FOXM1 in human ovarian cancer samples. Moreover, elevated expression of OTUB1 predicts poor prognosis in ovarian cancer. Indeed, it has been reported that OTUB1 could enhance breast cancer chemoresistance [35]. Based on the relationship between OTUB1 and FOXM1, and FOXM1's enhancement of ovarian cancer chemoresistance, [36] the effect of OTUB1 and the OTUB1-FOXM1 axis on ovarian cancer chemoresistance is a curious question for future studies. Interfering with OTUB-FOXM1 interaction could be an effective therapeutic method.

Previous studies have described other crucial genes targeted by OTUB1, such as SMAD2/3, ERα and c-IAP2, that could promote tumor progression [18, 19, 24]. Both ERα and c-IAP2 contribute to vascular formation through the NF-κB signaling pathway [37, 38], and SMAD2&3 are related to TGF-β signaling pathway, which drives cell invasion in somatic malignancies [39]. SMAD3 could be activated by FOXM1 to promote TGF-β dependent epithelial-mesenchymal transition (EMT) [25]. Thus, these downstream genes might form a network centered on OTUB1 that facilitates tumor invasion and metastasis through the NF-κB and TGF-β signaling pathways. Future studies might focus on the effect of OTUB1 on EMT through these two signaling pathways in somatic malignancies.

In summary, our study has revealed that OTUB1-FOXM1 interaction contributes to the tumorigenesis and aggression of ovarian carcinoma, and identified OTUB1 as a new biomarker for clinical prognostic prediction.

## MATERIALS AND METHODS

### Patient samples

A total of 225 ovarian cancer samples collected from the patients in Gynecological and Obstetrical Hospital of Fudan University were used in the study. None of the patients had received preoperative chemotherapy. The collected clinicopathological features included age, tumor size, staging, opposite ovary and fallopian tube involvement, lymph node and remote metastasis, and recurrence. All patients were staged based on the International Federation of Gynecology and Obstetrics (FIGO) staging system [40]. All follow-up information was collected by faculty of the department of pathology of Gynecology and Obstetrics Hospital of Fudan University, Shanghai, China. The follow-up interval was from the date of surgery to the date of death or the last clinical investigation. This study was approved by The Clinical Research Ethics Committee of Gynecology and Obstetrics Hospital of Fudan University. Written informed consent was obtained from all participants. For immunoblotting (IB), 25 fresh cancer tissue samples were ground on ice with tissue grinders (No. 357538, Fisher Scientific, USA) and centrifuged at 4°C for 10 min and quantified with the BCA quantification kit (Lot. 23225, Fisher Scientific, USA) followed by IB; for the immunochemistry (IHC), the other 200 enrolled samples were embedded with paraffin and sectioned consecutively. The clinicopathological information of these 200 and 25 cases are listed in Table 1 and the Supplementary Table 1, respectively.

### Cell culture, plasmids and reagents

Human ovarian carcinoma cell lines H293T, A2780, CAOV3, ES-2, HEY, OVCA433, HO8910, SKOV3, 3AO, and NIH: OVCAR3 were purchased from IBS Cell bank of Fudan University and Cell bank of Shanghai Institute, Shanghai, China. All cells were grown and maintained in either DMEM or RPMI-1640 medium (Lot. 12633-012, GIBCO, U.S.A.) supplemented with 10% fetal bovine serum (FBS, Lot.10099-141-FBS, GIBCO, USA) and 1% penicillin and streptomycin, maintain at 37°C in a humidified atmosphere with 5%CO_2_. Stable A2780 and SKOV3 cells infected with Lenti-OTUB1-IRES-EGFP, Lenti-OTUB1^ΔN15^-IRES-EGFP, Lenti-OTUB1^ΔN46^-IRES-EGFP and Lenti-OTUB1^A/S/A^-IRES-EGFP constructs were additionally grown in 4 μg/mL or 2 μg/mL Blasticidin S (Lot. A11139-02, Life, USA). The stable Lenti-shOTUB1 CAOV3 cell line transfected with Lenti-shOTUB1-GV112-GFP-Puro, Lenti-FOXM1 SKOV3 cells with Lenti-FOXM1-GV112-GFP-Puro, and Lenti-FOXM1^MutKEN^ SKOV3 cells with Lenti-FOXM1-GV112-GFP-Puro were maintained with 0.5 μg/mL puromycin (Lot. A1113803, Life, USA). The Lenti-OTUB1-shC and Lenti-OTUB1-shFOXM1 SKOV3 cells were maintained with both 2 μg/mL Blasticidin S and 0.5 μg/mL Puromycin. The information for specific plasmids and reagents are listed in the Supplementary Data.

### Immunochemistry

Consecutive paraffin sections of the 200 cases were prepared and incubated overnight at 37°C with primary antibodies against OTUB1 (HPA039176, Sigma Aldrich, USA) and FOXM1 (K-19, Santa Cruz) at a 1:50 dilution. OTUB1 antibody validations are shown (Supplementary Figure 2A). FOXM1 antibody was validated in our previous studies [6, 41]. Standard avidin-biotin immunohistochemical analysis of the sections was performed. The staining results were evaluated by at least three certified pathologists and the tumor cell staining was scored as follows: For FOXM1, 0 (no staining for nucleus despite of the staining result of cytoplasm or membrane), 1 (weak staining for nucleus [and cytoplasm] and area of positive staining <10%), 2 (moderate staining for nucleus [and cytoplasm] and area of positive staining among 10%–50%), and 3 (strong staining for nucleus [and cytoplasm] and area of positive staining >50%) [42, 43]; for OTUB1, 0 (no staining for cytoplasm or membrane), 1(weak positive for cytoplasm), 2(moderate positive for cytoplasm), and 3(strong positive for cytoplasm). The cases with score 0-1 were considered low and those with a score of 2-3 were considered high [42, 43].

### Mass spectrometer (MS) analyses

The FLAG-FOXM1 protein complexes were fractionated by SDS-PAGE, followed by Prussian blue staining. The Prussian blue-stained gel bands were treated with sequencing-grade trypsin (Lot. V5280, Promega, USA). The resulting peptides were analyzed by nano-HPLC-MS/MS with online desalting with a Famos autosampler. Electrospray ion trap MS was performed using an LTQ linear ion trap mass spectrometer (Thermo Finnigan). The fragment spectra were analyzed using the National Center for Biotechnology Information nonredundant protein database using Mascot (Matrix Science) and Sequest (Thermo Scientific).

### Protein purification, GST-pulldown, immunoprecipitation (IP), and immunoblotting (IB)

HIS-tagged OTUB1, OTUB1^ΔN15^, OTUB1^ΔN46^, OTUB1^ΔN170-271^, OTUB1^A/SA^, and OTUB1^C91S^ were expressed in *E. coli* and purified through a Ni-nitrilotriacetic acid (Lot. 30622, Qiagen) column or HIS-tagged magnetic purification beads (Millipore, USA) and eluted by 0.5 M imidazole.

Purified GST fusion protein was incubated with varied amounts of OTUB1 or FOXM1 fusion protein in binding buffer [25 mM tris(pH 8.0), 75 mM NaCl, 2.5 mM EDTA, 5 mM MgCl2, 2.5 mM DTT, 1% NP-40] at 37°C for 30 min. Glutathione sepharose 4B resin was equilibrated with binding buffer and 10 μL of resin was added to the incubated protein mixture and kept on a nutator for 30 min. Unbound protein fraction was separated from the resin by centrifugation at 3000×g for 3 min. Resin bound to the protein was washed with increasing concentrations of NaCl (100 mM to 400 mM) in binding buffer. Equal amount of 2×sample loading buffer [100 mM Tris-HCl (pH 6.8), 200 mM DTT, 4% SDS, 0.2% Bromophenol blue] was then added to the resin, boiled for 5 min, centrifuged briefly, and the supernatant was analyzed by SDS-PAGE. The protein bands were visualized by Coomassie blue staining as per standard procedures. Co-IP and immunoblotting were conducted as previously described [6]. 15% of cell lysates were used as input in the IP and co-IP assays.

### Immunofluorescence

Transfected A2780, SKOV3, and CAOV3 cells and negative control cells were fixed with 4% paraformaldehyde for 1h, penetrated with 0.5% Triton-100X (Sigma), and incubated with polyclonal rabbit anti-FOXM1 (1:50, Sigma), polyclonal rabbit anti-OTUB1 (1:50, Sigma), and monoclonal mouse anti-FLAG (1:400, Sigma). Cells were then stained with Alexa Fluor 546 (red) goat anti-rabbit alone or along with Alexa Fluor 488 (green) goat anti-mouse antibody (Invitrogen, U.S.A.) as well as DAPI (Invitrogen, U.S.A.) for DNA staining. Stained cells were analyzed under a Leica inverted fluorescence microscope with ProgRes Image Capture Software (JENOPTIK Optical System, Jena, German) and a Leica Confocal LAS-AF SP5 System.

### Ubiquitination/deubiquitination assays

For the *in vivo* ubiquitination/deubiquitination assays, cells were treated with MG132 (10 μM) for 3 hours and lysed. The lysates were immunoprecipitated by anti-MYC or anti-FOXM1 and immunoblotted by anti-ubiquitin. For the *in vitro* ubiquitination assays, purified FLAG-FOXM1 was incubated with 250 ng of Fraction II (F-340, BostonBiochem), 500 ng of ubiquitin (BostonBiochem) and 2μg of purified HIS-OTUB1 or mutants in 50 mM Tris HCl 8.0, 5 mM MgCl2, 1 mM ATP for 1 h at 37°C. The reaction was stopped by adding 1×SDS sample buffer, followed by SDS-PAGE. The protocol of the *in vitro* deubiquitination assays was in accordance with that in Sun et al. [22]

### RNA isolation and quantitative RT-PCR

Total RNA was extracted from tissue samples and cells using TRIzol (Invitrogen, Carlsbad, CA, USA) according to the manufacturer's protocol. The reverse transcription reactions were conducted using a PrimeScript^®^ RT reagent Kit (Takara, Dalian, China); the quantitative PCR reactions were then performed using SYBR^®^ Premix Ex TaqTM (Takara, Dalian, China), as previously described [44]. Glyceraldehyde-3-phosphate dehydrogenase (GAPDH) was included as the endogenous control to normalise the data. The primer sequences are listed in Supplementary Table 2.

### *In vitro* proliferation assays

Cell proliferation was indirectly assayed using the CCK-8 kit (Dojindo, Japan), which stains living cells. Approximately 5×10^3^ cells in 100μl were incubated in triplicate in 96-well plates. At 0, 24, 48, 72, and 96 h, the CCK-8 reagent (10μl) was added to each well and incubated at 37°C for 3h. The optical density at 450 nm was measured using an automatic microplate reader (Synergy4; BioTek, Winooski, VT, USA).

EdU imaging system was used to visualize and measure cell proliferation by counting the percentage of cells in progress of replication of DNA. 72 h after transfection, cells were incubated with 10 μM EdU solution for 2 h and fixed with 3.7% Formaldehyde (Sigma) and penetrated with 0.5% triton X-100 (Sigma) for 20 min. Cells were stained with EdU/Alexa Fluor Azide 594 for 30 min followed by Hoechst 33342(1:2000) for another 30 min and imaged at 100× and counted at 200× under the fluorescence microscopy (IX51, Olympus, Japan).

### *In vitro* metastasis assay

Transwell assay was used to assess cell invasion (Corning Co. Ltd., USA). The lower chambers were pre-coated with 100 μL Matrix gel (#354234, BD Bioscience, USA) for 30 min. 24 h after transfection, cells were seeded on the upper chamber at 3.0×10^4^/well in serum-free medium. Medium containing 20% fetal bovine serum medium was applied to the lower chamber as chemo-attractant. After 24 h incubation at 37°C, cells which invaded through the matrix gel and adhered to the lower surface of the filter were fixed with ethanol, stained with 0.5% crystal violet, photographed at 200×, and counted at 400× in 10 different fields to determine the average number of cells (BX51, Olympus, Japan).

### *In vivo* xenograft models

Animal experiments were approved by the Shanghai Medical Experimental Animal Care Commission. Female BALB/c-nu mice (4-5 weeks of age, 18-20 g) were maintained under specific pathogen-free conditions in the Experimental Animal Department of Fudan University. All of the experimental procedures involving animals were undertaken in accordance with the institute guidelines. 1×10^7^ Lenti-NC, Lenti-OTUB1, Lenti-OTUB1-shC, Lenti-OTUB1-shFOXM1, Lenti-OTUB1^ΔN46^, Lenti-OTUB1^A/S/A^, Lenti-FOXM1, Lenti-OTUB1-NC(FOXM1), Lenti-OTUB1-FOXM1, and Lenti-OTUB1-FOXM1^MutKEN^ stably infected SKOV3 cells were injected s.c. into the flank regions of 8 week old BALB/c female nude mice (n=8 per group) and allowed to grow for 24-30 days. All the mice were euthanized and the xenografts were excised out and measured. The tumor volumes were calculated using the formula 1/2×r1^2^×r2 (r1 < r2).

### Statistical analysis

Each experiment was performed in triplicate, and data are presented as the mean ±SD. All statistical analyses were performed using SPSS 20.0 (IBM, SPSS, Chicago, IL, USA). Student's t-test and one-way ANOVA were used in either 2 or multiple groups for statistical significance. Spearman rank order was used to analyze the correlations; DFS and DSS curves were calculated with the Kaplan-Meier method and were analyzed with the log-rank test. The DFS rate was calculated from the date of surgery to the date of progression (local and/or distal tumor recurrence) or to the date of death. The DSS rate was defined as the length of time between the diagnosis and death or last follow-up. Univariate analysis and multivariate models were fit using a Cox proportional hazards regression model. All tests were 2-sided, and P<0.05 was considered statistically significant.

Other methods (colony formation assay and wound-healing assay) used in this study are listed the Supplementary Information.

## SUPPLEMENTARY MATERIALS FIGURES AND TABLES


