# Supplementary material for: OTUB1-catalyzed deubiquitination of FOXM1 facilitates tumor progression and predicts a poor prognosis in ovarian cancer
Source: Oncotarget. 2016 May 4;7(24):36681–97. doi: 10.18632/oncotarget.9160 (PMC5095031; doi:10.18632/oncotarget.9160)
Supplement: Supplementary file 1 [file oncotarget-07-36681-s001.pdf]

SUPPLEMENTARY MATERIALS AND METHODS

Antibodies and reagents

For antibodies used: HA (H9658), FLAG (M2 monoclonal), OTUB1 (HPA039176) and FOXM1 (HPA029974) were from Sigma Aldrich, St. Louis, MO, USA; MYC (mouse monoclonal 9E10) and FOXM1 (K-19 polyclonal and E-8 monoclonal) were from Santa Cruz Biotechnology, Dallas, Texas, USA; HIS (#2365), GAPDH (#5174), SP1 (#9389), ubiquitin (#3933S), K48-specific (#4289) and K63-specific ubiquitin (#5621) were from Cell Signaling, Boston, Massachusetts, USA; K11-specific ubiquitin (MABS107-1, Merck Millipore, MA, USA). For reagents used: Lipofectamine 2000 transfection reagent (Lot. 11668-027 Invitrogen, Carlsbad, CA, USA). MG132 and cycloheximide (CHX) (Lot. C2211 and Lot. C7698, Sigma). RIPA lysis buffer (Lot. 89901, Thermo Scientific). Blasticidin S (Lot. Ant-bl-5, Invitrogen, Life technology, USA). Puromycin (P7130-1G, Sigma, USA).

Plasmids and stable-transfected cell lines

Full-length human OTUB1 (OTUB1 isoform1, NM_017670.2) and FOXM1 (FOXM1 isoform 3, NM_202003.2) cDNAs were amplified from H293T cell mRNA by RT–PCR and cloned into pcDNA3.1-OTUB1-GFP, pcDNA3.1-FLAG-OTUB1, pcDNA3.1-HA-OTUB1, pcDNA3.1-FOXM1-GFP, pCMV-FoxM1-MYC, pCMV-HA-FOXM1 and 3xFLAG-FOXM1, the primers for OTUB1 and FOXM1 were listed in Supplementary Table 2. The pcDNA3.1-Ubiquitin-HA plasmid was obtained from Dr. Q.W. of Nanjing Normal University. The K48only-Ubquitin-HA, K11only-Ubquitin-HA and K63only-Ubiquitin-HA plasmids were chemically synthesized first (Generay Biotech. Co. Ltd., Shanghai, China) then cloned into the pcDNA3.1 vector. All N-terminal FLAG-tagged OTUB1 deletion mutants and N-terminal FLAG-tagged FOXM1 deletion mutants were generated by PCR and cloned into pcDNA3.1–FLAG or 3xFLAG-MYC vector, and the primers were listed in Supplementary Table 2. All OTUB1 plasmids with point mutations and the mutant FOXM1 with 207K208E209N converted to Alanine were constructed using site-directed mutagenesis (Stratagene, Lot: 210518). The siRNAs and shRNAs of OTUB1 were purchased (Catalog: 31334-31336, GeneChem. Co. Ltd., Shanghai, China). The siRNA and shRNA of FOXM1 were purchased (Catalog: 28550-1, GeneChem. Co. Ltd., Shanghai, China). The targeting sequences of OTUB1-shRNA and FOXM1-shRNA were listed in Supplementary Table 2.

Colony formation assay

The stable OTUB1-expressing or knockdown cells and their control counterparts were seeded in 60mm dishes or six well plates at the density of 1000 cells, and the plates were incubated at 37°C for 14 days. The colonies were visualized by the crystal velvet blue staining.

Wound-healing assay

The *in vitro* wound-healing assay was used to assess cell motility. Transfected cells were plated at equal density in 6-well plates and grown to confluence. Wounds were then generated with a sterile pipette tip, cells were rinsed two times with PBS and serum-free culture medium was added. Photos were taken at 24 h at 40× under microscope (BX51, Olympus, Japan).

Primer sequences for RT-qPCR

The primers for the mentioned genes in RT-qPCR in the article were listed as follows:

5’-GAGGTTTTTCTGTGGTTGT-3'(forward) and 5'-GCTCAGGAGTAGATAGGTG-3' (reverse) for OTUB1;

5’-AACTCCATCCGCCACAACC-3’(forward), 5’-GCTTAAACACCTGGTCCAATGTC-3’(reverse) for FOXM1.

5’-GCCTCGTCTCATAGACAAGATGG-3’ (forward) 5’-CTCAGTATCCTTGCTGGGCTG-3’ (reverse) for GAPDH.

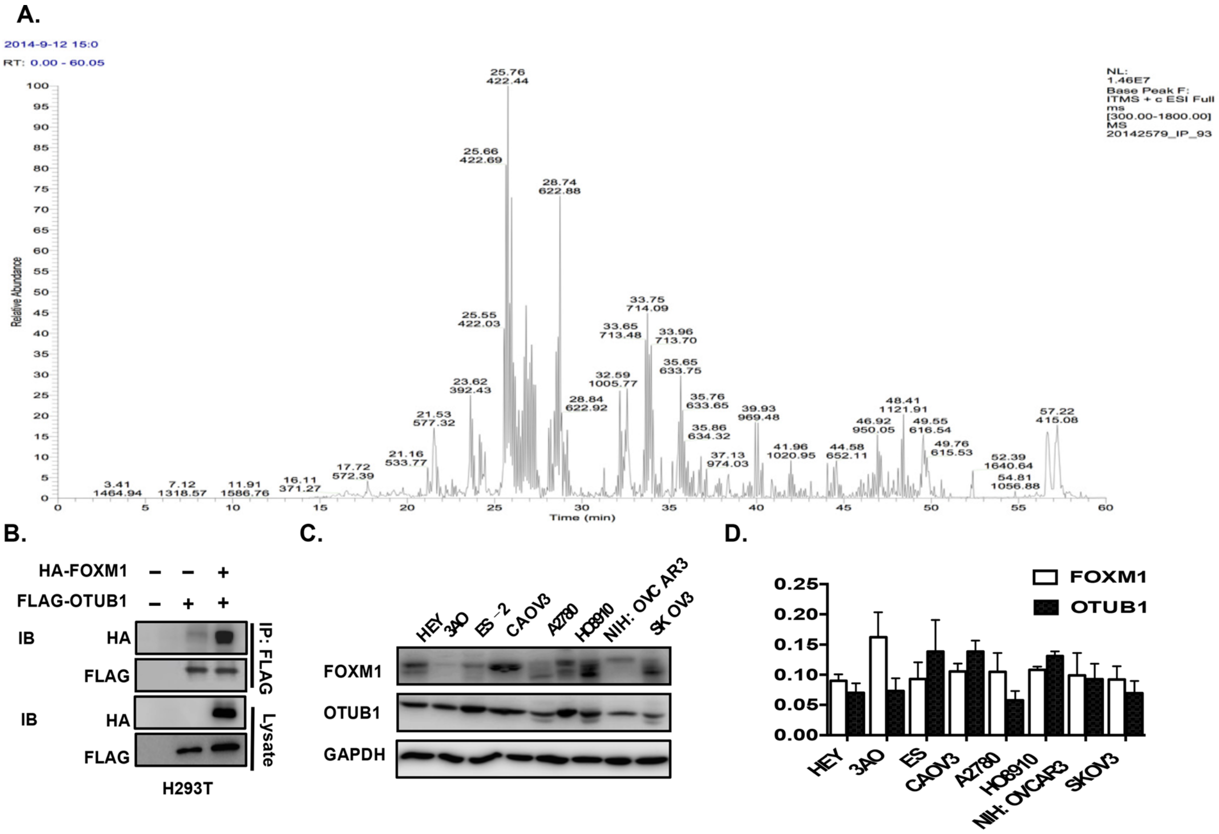

Supplementary Figure S1: Identification of OTUB1 as a FOXM1 binding target. A. OTUB1 was identified in the 3xFLAG-FOXM1 IP complex immunoprecipitated using anti-FLAG antibody from the H293T cells transfected with FLAG-FOXM1. The protein bands of SDS-PAGE were cut and digested and subjected to MS LC-MS/MS analysis. The base peak spectra of all the binding targets of FOXM1 are shown. **B.** H293T cells were transfected with HA-FOXM1 and/or pcDNA3.1-FLAG-OTUB1. The cell lysates were subjected to IP using anti-FLAG antibody followed by IB with the anti-HA antibody. **C.** The baseline expression of OTUB1 in 9 ovarian tumor cell lines by IB. **D.** The baseline mRNA levels of OTUB1 in 9 ovarian tumor cell lines by RT-qPCR.

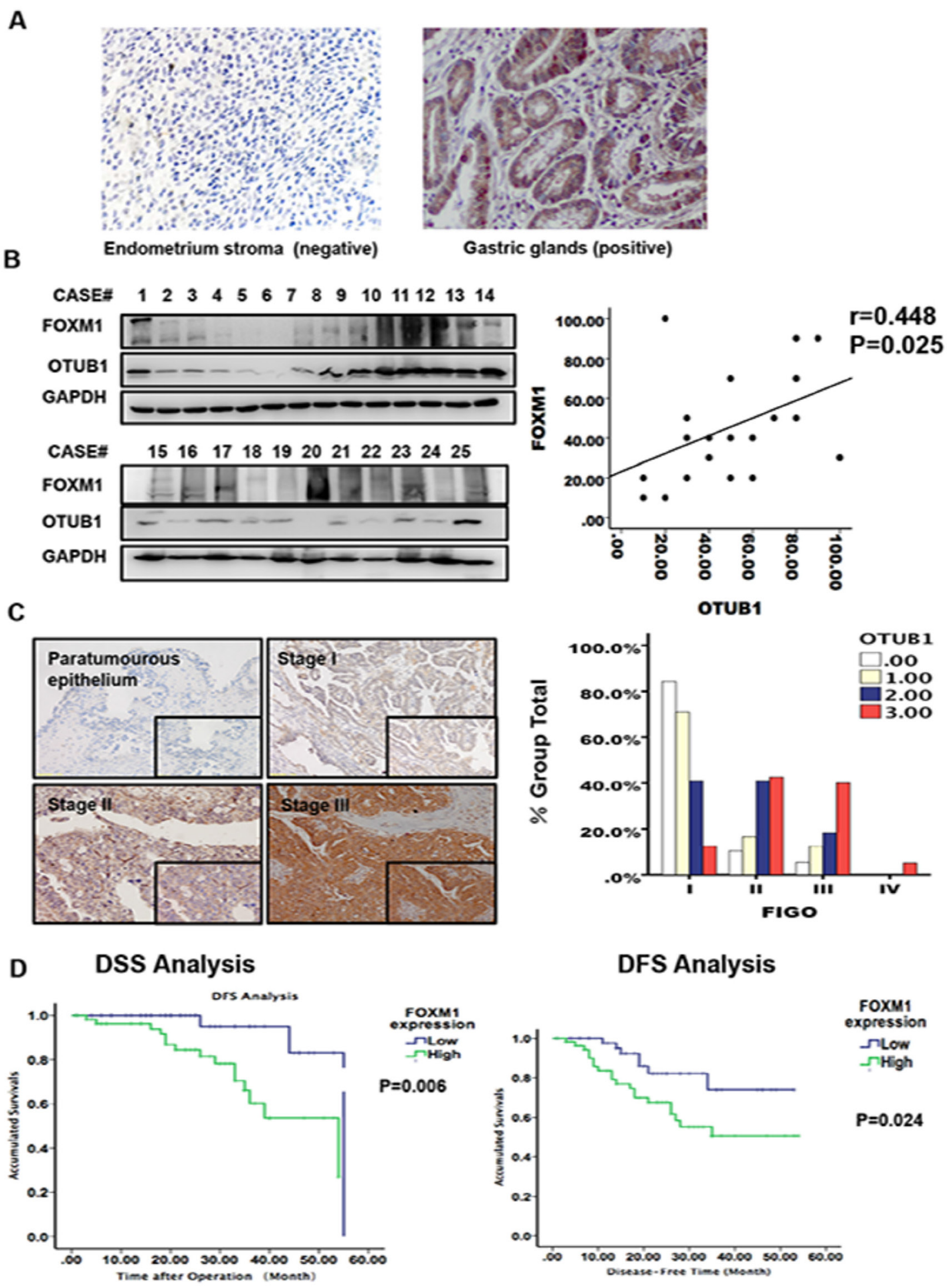

Supplementary Figure S2: OTUB1 is clinically correlated to FOXM1 and predicts poor prognosis in ovarian carcinoma. **A.** The outside controls according to the manufacturer's instructions (Sigma Aldrich, USA) of OTUB1 in immunochemistry by anti-OTUB1 antibodies (HPA039176, Sigma Aldrich). Left: Negative control (endometrium stroma). Right: Positive control (gastric gland). **B.** Immunoblotting results of FOXM1 and OTUB1 in 25 tissues of ovarian carcinoma were shown. The integrated optical intensities of immunoblotting were scanned by software ImageJ and analyzed with Pearson correlation (*r*=0.448, *p*=0.025) **C.** Representative images of OTUB1 expression in paratumorous epithelium and ovarian carcinoma of Stage I, II and III. The percentage of immunochemistry scores of OTUB1 in different FIGO stages were calculated and graphed. **D.** Kaplan-Meier disease-free survival (DFS) and disease-specific survival (DSS) curves of patients with different expressions of FOXM1 in ovarian cancer (Low vs. High).

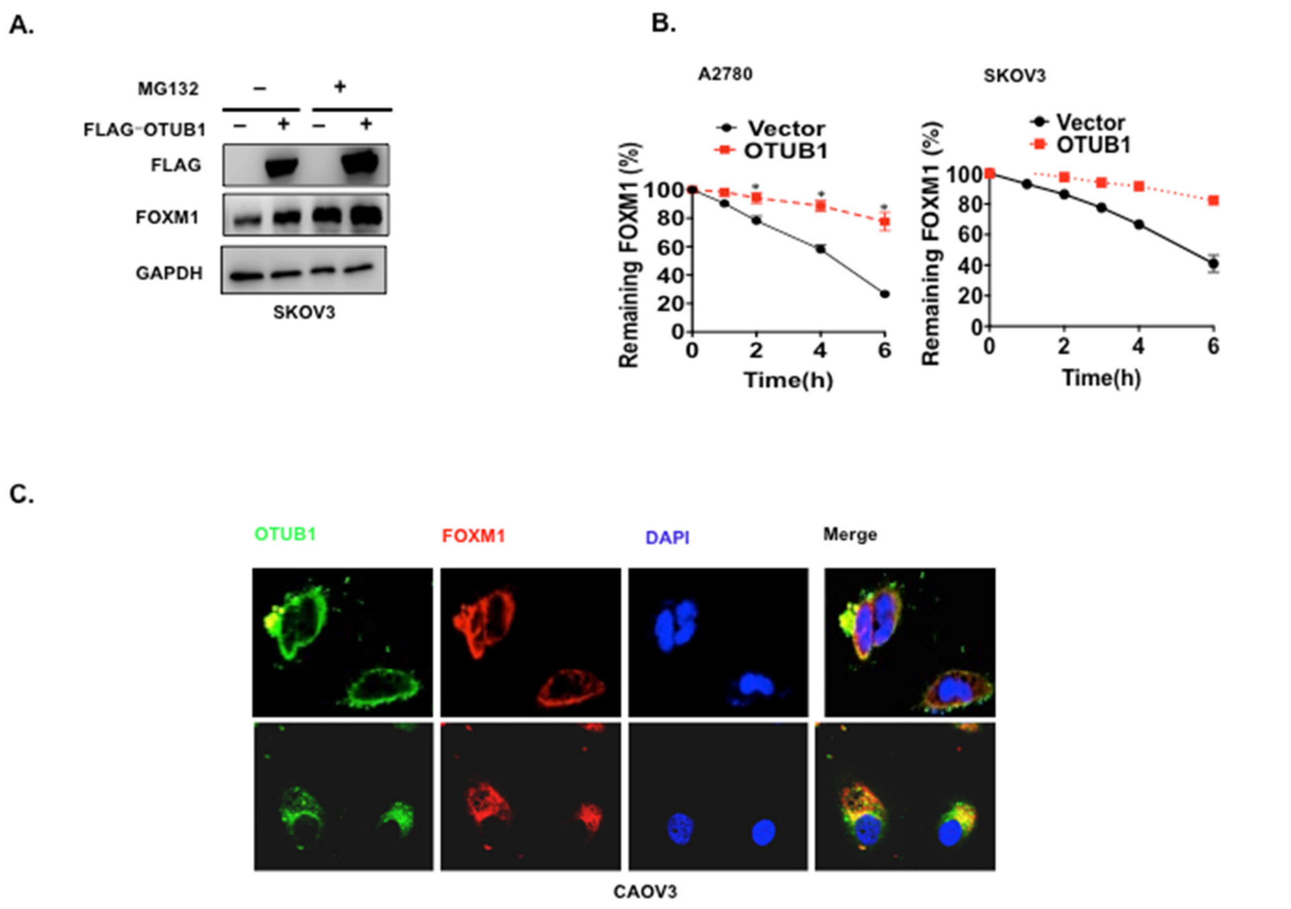

Supplementary Figure S3: OTUB1 stabilizes FOXM1 in cells. A. SKOV3 cells were transfected with or without FLAG-OTUB1 for 48h, and MG132 (10 μM) was added for 3h. Cell lysates were subjected to IB analysis. **B.** The average remaining FOXM1 in Figure 2 were quantified by software Image J and graphed. The experiments were performed triplicates. *: *p*<0.01. **C.** CAOV3 cells were fixed and penetrated with 0.5% Triton-X100 and incubated with anti-FOXM1 (Rabbit, red) and anti-OTUB1 (Mouse, green) overnight. DAPI was used for the nucleus staining.

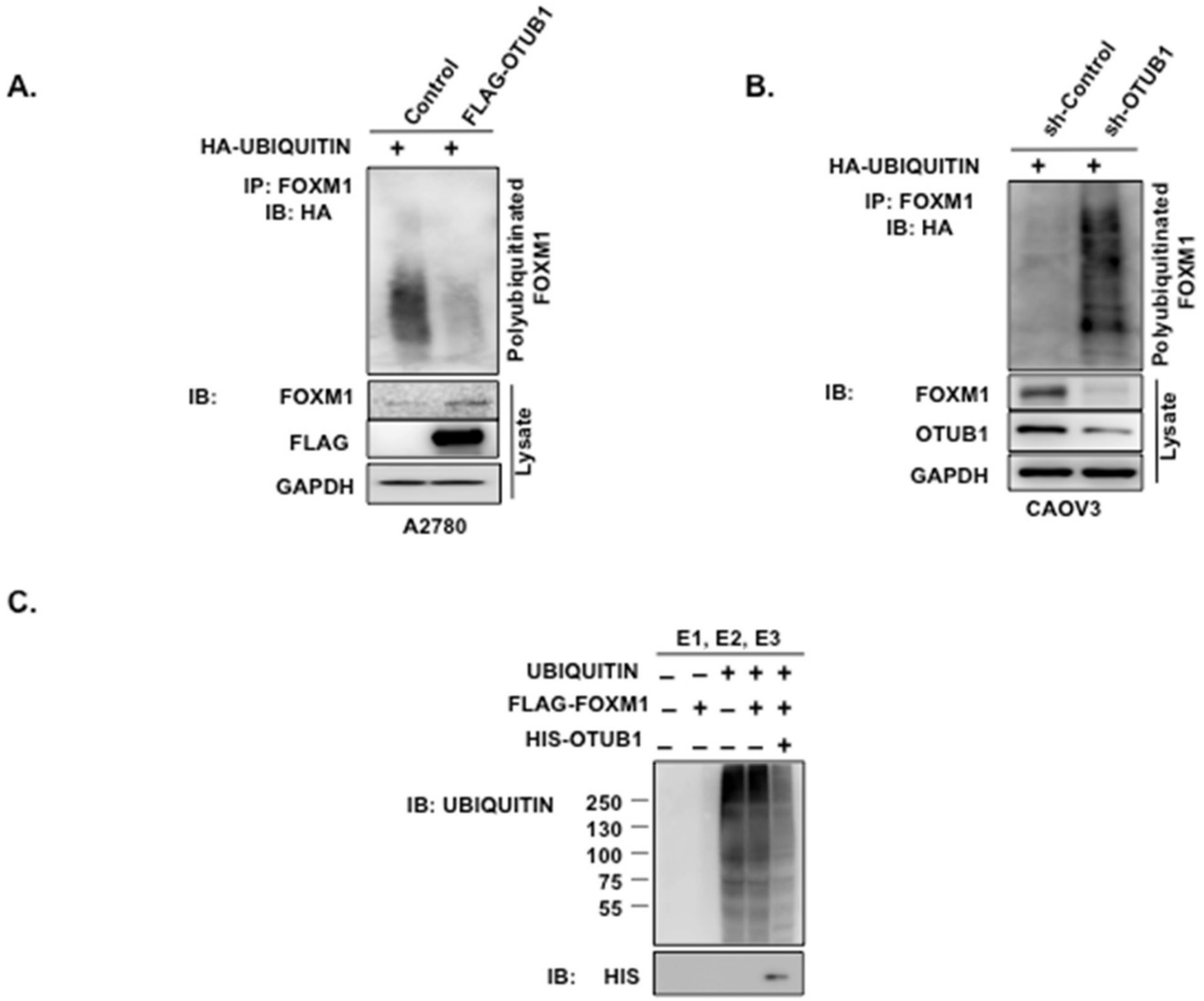

Supplementary Figure S4: OTUB1 suppresses FOXM1 ubiquitination *in vivo* and *in vitro*. A. The *in vivo* ubiquitination assay indicated that overexpression of OTUB1 inhibited FOXM1 ubiquitination in A2780 cells. **B.** The *in vivo* ubiquitination assay showed that knockdown of OTUB1 led to accumulation of ubiquitinated FOXM1 in CAOV3 cells. **C.** The *in vitro* ubiquitination assay suggested that purified HIS-OTUB1 protein effectively suppressed FOXM1 ubiquitination *in vitro*.

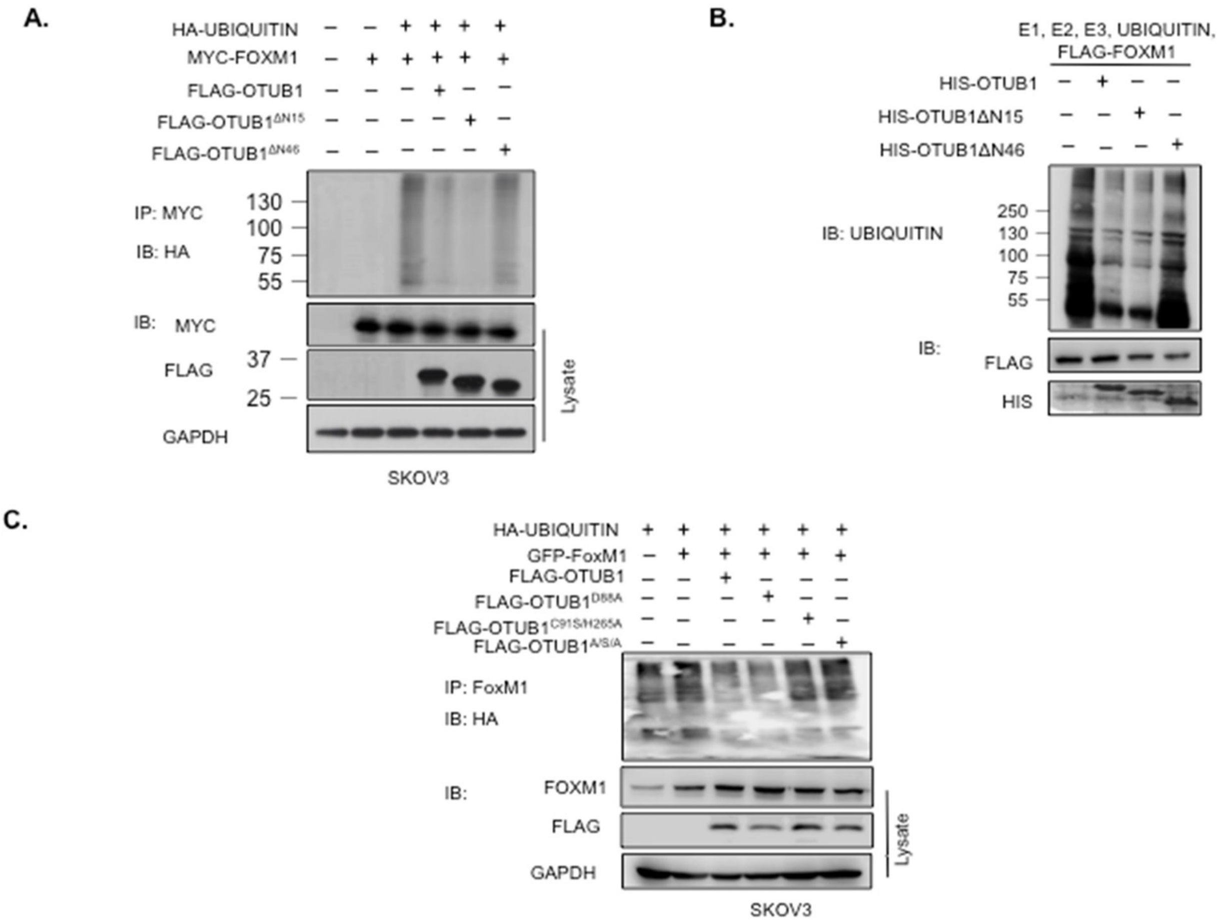

Supplementary Figure S5: OTUB1-mediated FOXM1 deubiquitination requires the binding of key domains of OTUB1 to FOXM1. A. The immunoblotting result of the *in vivo* ubiquitination; OTUB1$^{\Delta N46}$ but not OTUB1$^{\Delta N15}$ drastically lost DUB activity on FOXM1. **B.** The immunoblotting result of the *in vitro* ubiquitination; OTUB1$^{\Delta N46}$ but not OTUB1$^{\Delta N15}$ drastically lost DUB activity on FOXM1. **C.** The *in vivo* ubiquitination assay showed that OTUB1$^{A/S/A}$ but not OTUB1^{D88A} or OTUB1$^{C91S/H265A}$ lost the DUB activity to suppress FOXM1 ubiquitination in SKOV3 cells. OTUB1$^{A/S/A}$: OTUB1 $^{D88A/C91S/H265A}$.

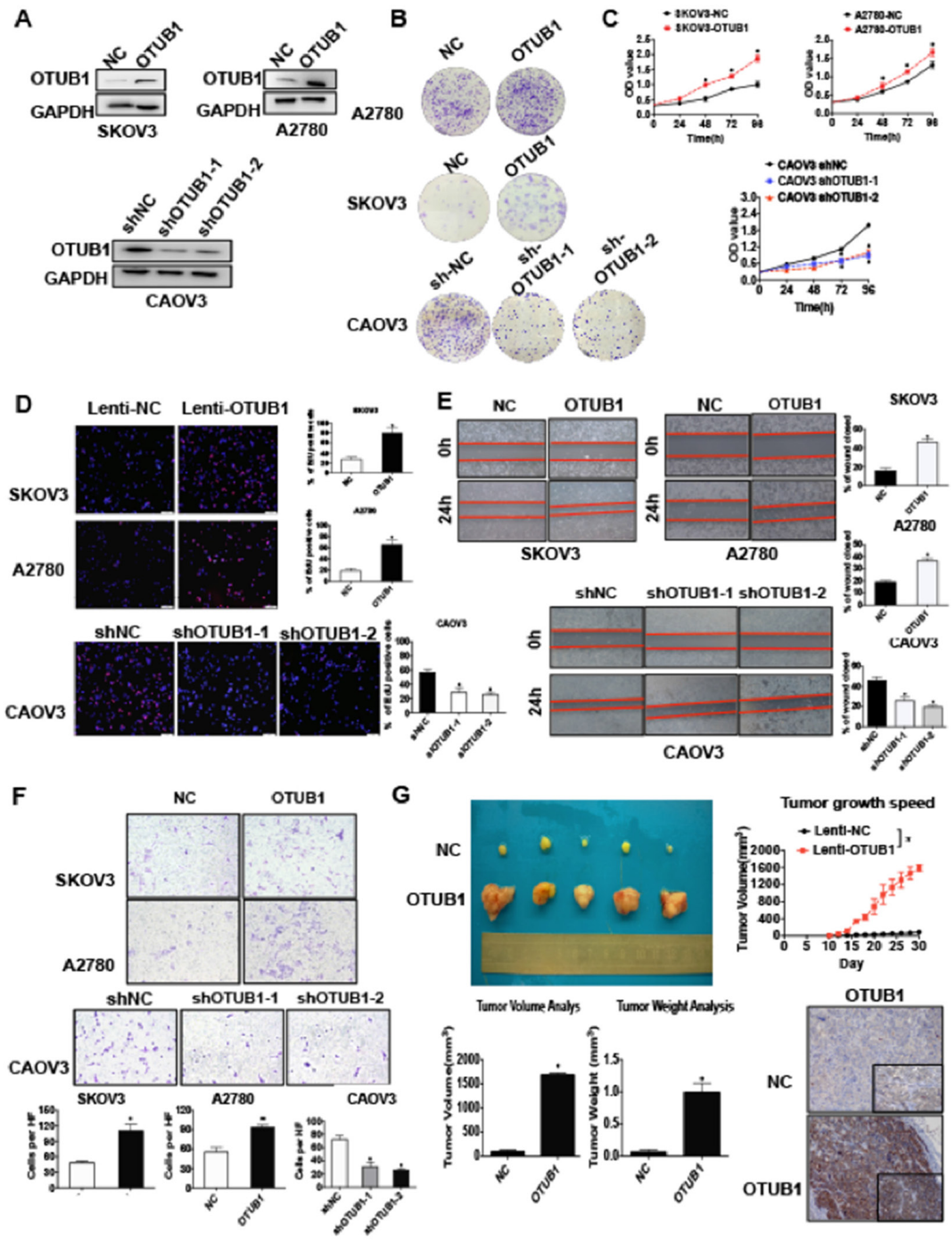

Supplementary Figure S6: OTUB1 promotes tumorigenesis and tumor progression in ovarian carcinoma. A. The immunoblotting results showed the efficiencies of overexpression of OTUB1 in SKOV3 and A2780 and knockdown of OTUB1 in CAOV3 by shRNA1 and 2. **B.** The colony-forming results of overexpression of OTUB1 in A2780 and SKOV3 and knockdown of OTUB1 in CAOV3 cells. **C.** The CCK8 counting assay showed that the cell proliferation was stimulated by overexpression of OTUB1 in SKOV3 and A2780 while inhibited by knockdown of OTUB1 in CAOV3. *: *p*<0.01. **D.** Representative images of EdU immunofluorescence assay in OTUB1-overexpressing SKOV3 and A2780 and in CAOV3 with shOTUB1. The percentage of EdU positive cells were graphed under 100× and calculated under 200×. *: p<0.01. **: p<0.05. **E.** The wound-healing result of overexpression of OTUB1 in A2780 and SKOV3 and knockdown of OTUB1 in CAOV3 cells. *: *p*<0.01. **F.** The representative images of transwell assay conducted in OTUB1-overexpressing SKOV3 and A2780, and in CAOV3 with shOTUB1. The penetrated cells were graphed at 200× and counted under 400× magnification. *: p<0.01. **G.** The xenograft results of NC and OTUB1 SKOV3 cells. Tumors were photographed and the speed of tumor growth was illustrated as curves. The volume and weight of tumors were calculated and analyzed. *: *p*<0.01. Immunochemistry was performed with anti-OTUB1 antibody to show the efficiency of OTUB1 overexpression.

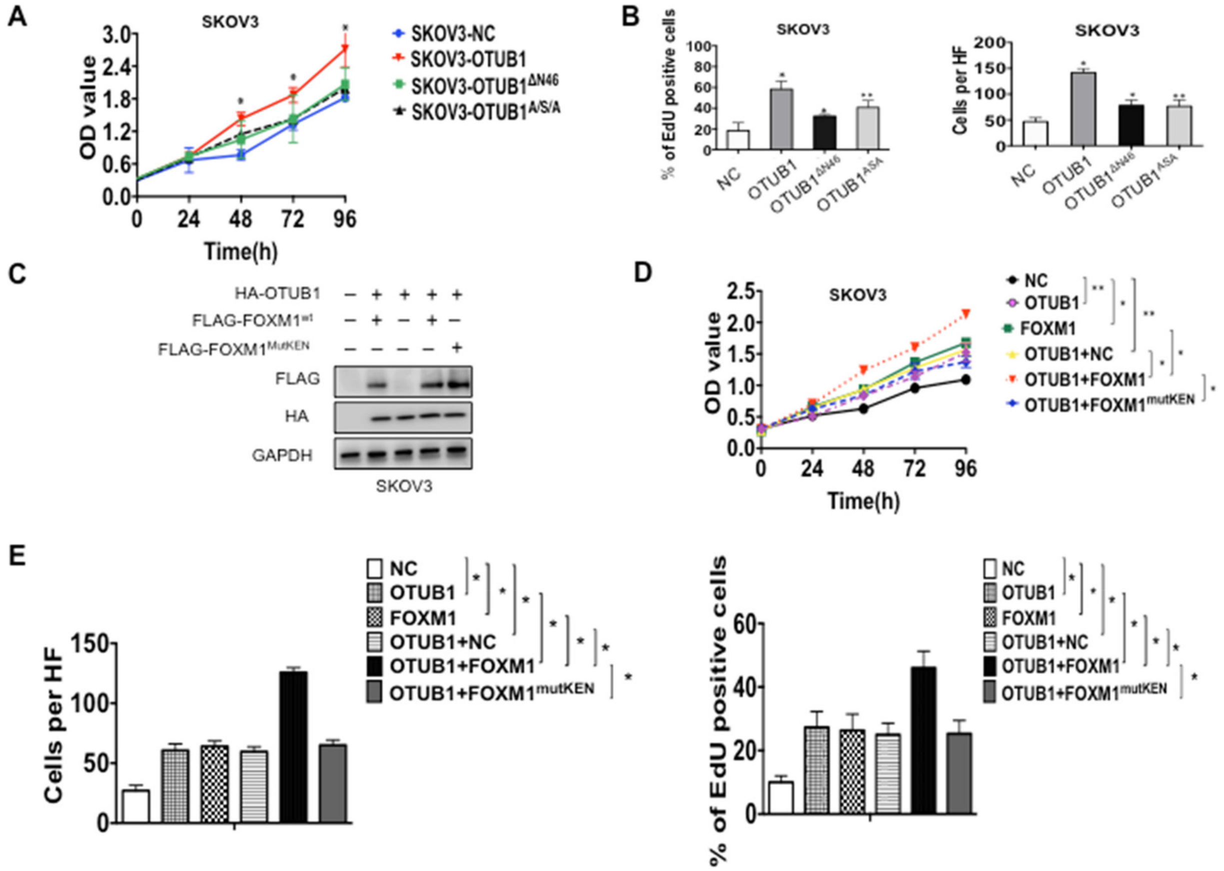

Supplementary Figure S7: FOXM1 deubiquitination is the core function of OTUB1. A. The CCK8 results of NC, OTUB1, OTUB1$^{\Delta N46}$ and OTUB1$^{A/S/A}$ SKOV3 cells. *: $p<0.01$. **B.** The statistical graphs of EdU (B1) and Transwell (B2) assays in NC, OTUB1, OTUB1$^{\Delta N46}$ and OTUB1$^{A/S/A}$ SKOV3 cells. *: $p<0.01$. **: $p<0.05$. **C.** The immunoblotting results of SKOV3 cells transfected with vector, OTUB1, FOXM1, OTUB1-NC (FOXM1), OTUB1-FOXM1 and OTUB1-FOXM1^{MutKEN}. **D.** The CCK8 results of NC, OTUB1, FOXM1, OTUB1-NC (FOXM1), OTUB1-FOXM1 and FOXM1^{MutKEN} SKOV3 cells. *: $p<0.01$. **: $p<0.05$. **E.** The statistical graphs of EdU and Transwell assays in NC, OTUB1, FOXM1, OTUB1-NC (FOXM1), OTUB1-FOXM1 and FOXM1^{MutKEN} SKOV3 cells. *: $p<0.01$. **: $p<0.05$.

Supplementary Table S1: The clinicopathological parameters of 25 tissue donors

Clinicopathological Features		n	%
**All cases**		25	100
**Age (years)**	<40	15	75.00
**(Average 57.6)**	≥40	10	25.00
**Tumor size**	<5cm	11	44.00
	≥5cm	14	56.00
**Histological subtypes**	Serous	10	40.00%
	Endometroid	6	24.00%
	Mucinous	0	0.00%
	Clear cell	9	36.00%
**FIGO Stage**	I	11	44.00
	II	7	28.00
	III, IV	7	28.00
**Opposite ovary involved**	-	13	52.00
	+	12	48.00
**Tumor cells in peritoneal fluid**	-	7	28.00
	+	18	72.00
**Fallopian tube involved**	-	11	44.00
	+	14	56.00
**Peritoneal implants**	-	10	40.00
	+	15	60.00
**Lymph node metastasis**	-	17	68.00
	+	8	32.00
**Remote metastasis**	-	24	96.00
	+	1	4

Supplementary Table S2: Primer Sequences of plasmid and Targeting Sequences for siRNA or shRNA

Gene	Primer Sequences and Targeting Sequences for siRNA or shRNA
OTUB1	5'-CCCCAAGCTTACCATGGACTACAAGGACGACGATGATAAGATGGCGGC GGAGGAACCT-3'(forward)
	5'-CGCGGATCCCTATTTGTAGAGGATATCGTAGTGT-3'(reverse)
FOXM1	5'-AACAAGCTTATGAAAACTAGCCCCCGTCGG-3' (forward)
	5'-AAAGGATCCCTACTGTAGCTCAGGAATAAAC-3' (reverse)
OTUB1$^{\Delta N15}$	5'-CCCAAGCTTACCATGGACTACAAGGACGACGATGATAAG AGCGACTCCGAAGGTGT-3'(forward)
	5'-CGCGGATCCCTATTTGTAGAGGATATCGTAGTGT-3'(reverse)
OTUB1$^{\Delta N46}$	5'-CCCAAGCTTACCATGGACTACAAGGACGACGATGATAAG CTGGTGTCAGAGCGGCT-3'(forward)
	5'-CGCGGATCCCTATTTGTAGAGGATATCGTAGTGT-3'(reverse)
FOXM1$^{\Delta N30}$	5'-AACAAGCTTGAACCTAAGAGATCCCCTGCC-3'(forward)
	5'-AAAGGATCCCTACTGTAGCTCAGGAATAAAC-3' (reverse)
shOTUB1-1 (siOTUB1-2)	CCACCAATCCGCACATCTT
shOTUB1-2 (siOTUB1-3)	TTTCTATCGGGCTTTCGGA
siOTUB1-1	GAGTATGCTGAAGATGACA
Scramble	TTCTCCGAACGTGTCACGT
shFOXM1 (siFOXM1)	CCAACAGGAGTCTAATCAA

Supplementary Table S3: The major identified protein list of FLAG-FOXM1 by LS/MS

number	Protein name	Cover Percent Diff(MH+)
1	Heterogeneous nuclear ribonucleoprotein U OS=Homo sapiens GN=HNRNPU PE=1 SV=6	17.58%
2	Myosin-10 OS=Homo sapiens GN=MYH10 PE=1 SV=3	9.22%
3	Ubiquitin thioesterase OTUB1 OS=Homo sapiens GN=OTUB1 PE=1 SV=2	18.35%
4	Heterogeneous nuclear ribonucleoproteins A2/B1 OS=Homo sapiens GN=HNRNPA2B1 PE=1 SV=2	17.00%
5	Heat shock cognate 71 kDa protein OS=Homo sapiens GN=HSPA8 PE=1 SV=1	10.22%
6	X-ray repair cross-complementing protein 6 OS=Homo sapiens GN=XRCC6 PE=1 SV=2	13.39%
7	Scaffold attachment factor B1 OS=Homo sapiens GN=SAFB PE=1 SV=4	10.53%
8	Myosin-9 OS=Homo sapiens GN=MYH9 PE=1 SV=4	10.63
9	Heterogeneous nuclear ribonucleoprotein K OS=Homo sapiens GN=HNRNPK PE=1 SV=1	12.07%

Supplementary Table S4: Univariate and Multivariate analysis of Disease-free survival in ovarian cancer

Variables	Categories	Univariate analysis		P value	Multivariate analysis		P value
		HR	95%CI		HR	95%CI	
Age	<40/≥40	1.036	0.464-2.316	0.931	--	--	--
FIGO	I/II-IV	2.533	1.544-4.156	<0.001*	--	--	--
Tumor size	<5cm/≥5cm	0.722	0.318-1.637	0.435	--	--	--
Lymph node metastasis	-/+	7.371	3.255-16.689	<0.001*	6.472	1.387-30.198	0.017*
FOXM1 expression	Low/High	6.822	2.326-20.013	<0.001*	--	--	--
OTUB1 expression	Low/High	11.445	2.681-48.860	0.001*	6.570	1.301-33.181	0.023*

HR, hazard ratio, 95%CI, 95% confidence interval
*: P<0.05

Supplementary Table S5: Univariate and Multivariate analysis of Disease-specific survival in ovarian cancer

Variables	Categories	Univariate analysis		P value	Multivariate analysis		P value
		HR	95%CI		HR	95%CI	
Age	<40/≥40	1.120	0.641-1.957	0.689	--	--	--
FIGO	I/II-IV	3.065	2.147-4.377	<0.001*	1.975	1.165-3.347	0.012*
Tumor size	<5cm/≥5cm	1.183	0.663-2.111	0.568	--	--	--
Lymph node metastasis	-/+	4.067	2.236-7.399	<0.001*	--	--	--
FOXM1 expression	Low/High	6.394	3.098-13.199	<0.001*	2.235	1.016-4.913	0.045*
OTUB1 expression	Low/High	12.402	4.443-34.620	<0.001*	4.443	1.433-13.772	0.010*

HR, hazard ratio, 95%CI, 95% confidence interval
*P<0.05.
